# Older adults’ motivations to participate or not in epidemiological research. Qualitative inquiry on a study into dementia in Switzerland

**DOI:** 10.1371/journal.pone.0247141

**Published:** 2021-02-12

**Authors:** Maddalena Fiordelli, Marta Fadda, Rebecca Amati, Emiliano Albanese

**Affiliations:** Institute of Public Health, Faculty of Biomedical Sciences, Università della Svizzera italiana, Lugano, Switzerland; Universitat Luzern, SWITZERLAND

## Abstract

**Introduction:**

High participation in epidemiological studies is crucial for both external and internal validity. Because response rates have declined in recent years, there is an increasing need to understand the drivers and the barriers to research participation. This study aims to uncover the motivations in favour and against participation of older adults to an epidemiological study on health and dementia.

**Methods:**

Twenty-two older adults, who already took part to the preliminary phase of an epidemiological study in Switzerland, agreed to participate to semi-structured, face-to- face interviews. An experienced researcher carried out all interviews in a quiet place of choice of the interviewee either at their domicile or the university, between November 2019 and January 2020. The interviews were audio and video taped, transcribed verbatim, and thematically analysed by two independent researchers.

**Results:**

We identified three main themes for the motivations in favour of participation (i.e. personal, related to the outcomes of research, and altruistic motivations), and we highlighted subthemes for each theme (e.g. personal motivations: curiosity; civic engagement; interest in the topic; trust in science; everyone counts; openness; play the game). Motivations against participation reflected the first two themes, while there was no counterpart for altruistic motivations.

**Conclusions:**

Our thematic analysis revealed that older adults hold specular motivations in favour and against participation to research. Studying jointly motivations in favour and against provides information for recruitment strategies and to overcome barriers to participation, respectively. Participatory action research can inform the design and conduction of and should precede epidemiological studies in older adults, and can potentially contribute to attain high response rates.

## Introduction

The exclusion of some subgroups of the population (i.e. pregnant women, children, elderly people) is common by design [1] and for ethical reasons [2, 3] in clinical and experimental research. Those who we seek to include in the study, the study sample, are drawn from the source population, which is the accessible (and available) sub-set of the target population to which results may generalize. While sampling designs and techniques have improved over the past decades [4, 5], the proportion of those in the planned sample who actually participate in epidemiological studies has steadily declined in western countries [6]. Because the representativeness of study samples is key in descriptive epidemiology, and low participation rates can introduce bias and compromise both external and internal validity of results, there is an increasing interest in studying the modulating factors of participation in epidemiological research.

Societal and cultural changes on the one hand, and the increasing complexity of research methods over the past decades have contributed to decreasing participation rates in both clinical-epidemiological and translational research [7–9]. Moreover, older adults are often excluded by design [1], and/or for ethical reasons [3]. However, indirectness of evidence is increasingly problematic also because of the demographic ageing of society [8]. Understanding the factors that modulate participation in research is important [10], especially for studies that focus on older adults [11]. The differences between older adults who participate and do not in research have been explored using both quantitative [12–14], and qualitative approaches [10, 15–18]. The latter proved to be particularly suitable in uncovering both the drivers to and the experience of participation [10]. Beyond personal benefits [10], altruistic motivation was a main behavioural driver in particular for participation in genetic [19], epidemiological studies [8, 10, 12], and prevention trials, including on dementia [13] and memory research [17]. Contributing to a broad societal benefit was one of the main reason for older adults to participate in clinical research, including on Alzheimer’s disease [20]. Conversely, qualitative studies with African Americans older adults suggested that reasons for non-participation may include (a) mistrust, (b) avoidance and fear of acknowledging problems, and (c) seeing the risks [16]. However, no qualitative study so far has systematically reflected upon the reasons for both participation and non-participation in population health studies of older adults.

Reasons in favour and against participation of older adults are best studied jointly to inform communication and recruitment strategies for increasing participation rates in population studies [21]. The aim of this study was to shed the light on the motivations in favour and against participation of older adults to an epidemiological study on health and dementia.

## Materials and methods

### Study design

Between December 2019 and January 2020, we conducted a series of semi-structured interviews levering a pilot and validation study of the procedures and methods for a large population-based study on dementia and its impact in southern Switzerland. The sample of the validation study was a convenience sample, and participants were volunteers. It included dementia patients as well as cognitively healthy participants. Dementia patients were recruited from the local memory clinic, healthcare and daycare services for older adults (e.g. geriatric, neurologic, and psychiatric). Cognitively healthy participants were recruited through standard advertisement and word of mouth through local older adults’ associations. During the validation study we conducted dyadic interviews of study participant with an informant (i.e. a person who is in close contact and knows well the interviewee). Both participants and informants were recruited for the current study. We chose semi-structured interviews to allow the individuals to express their experiences and motivations with their own words.

### Study participants

All participants (n = 200 older adults 65+) to the validation study agreed to receive qualitative interviews besides the cognitive tests, but only a minority (n = 35) provided contact information. They were later recruited via email or telephone for a semi-structured interview.

### Ethics, consent and permission

We recruited participants after informed signed consent. For people with dementia who lacked capacity to express consent, the informed consent was addressed to a relative or his/her legal representative. In cognitively healthy participants, the capacity to consent was determined by the interviewer using standard, semi-structured questions when checking for the eligibility criteria: being ≥ 65 years old and having an informant. The study was approved by the Cantonal Ethical Committee in the scope of the broader epidemiological study. Before the interview started the interviewer read a release form for the video material collected and its future uses, and the interviewee signed it in two copies.

### Data collection

We designed and conducted semi-structured interviews based on a predefined interview grid developed for this study and covering the following research topics: experience of participation in the validation study, opinion on the return of general and individual-specific research findings, and informed consent (S1 Appendix). After the first two interviews, we expanded the interview guide to gather more information on motivations in favour and against participation. First, we carried out simulated dialogues in which the interviewees were asked to persuade the interviewer to participate in the epidemiological study; second, we discussed and asked opinions on possible communication messages and channels for involving the population in the study.

An experienced interviewer (RA) carried out all interviews in a quiet place of choice of the interviewee either at their domicile or the university. The interviews lasted on average 45 minutes. Socio-demographic data were collected after the interview. The interviews were transcribed verbatim and each transcription was anonymized and attributed a unique identifier. We conducted interviews until data saturation was reached and confirmed, and sample size was defined *a posteriori* accordingly [22].

### Data analysis

Two independent researchers (MF* and RA) analysed the transcripts of the interviews and held regular meetings to discuss and harmonize their coding. They used an inductive thematic approach to analyse the data and identify patterns of themes: familiarizing with the content of the transcripts, highlighting meaningful quotes regardless of their length, condensing them under a number of labels, organizing the generated labels hierarchically, creating relationships between them, and identifying remarkable quotations to represent thematic similarities, differences, and contradictions [23]. A map highlighting themes, subthemes and relationships was created. Anonymized quotes served to represent each of the themes and subthemes. The analysis aimed at identifying factors hindering or favouring participation, and foreseen benefits and their valence. We focused on eliciting granular information on expectations, possible barriers, and facilitators of successful recruitment.

## Results

Six participants asked to be interviewed in couples. Thirteen of the nineteen interviews were video recorded by a professional video maker, while the first six were video recorded by the same researcher (RA). RA constantly reflected on her role in the interview encounter and naturally established a rapport with participants that boosted the richness of the data collected [24].

### Participants

Twenty-two individuals (Table 1; male = 11) agreed to participate in the present study. The majority of them (n = 20) accepted to be video and audio recorded during the in-depth interviews, while two gave consent only to be audio taped. Six participants were informants (spouse = 4; daughter = 2) of individuals with dementia. The mean age of participants was 71 years (SD: 9.3; min 45 –max 86). More than half of the participants were living in the urban district (n = 13), and the rest lived in rural areas. Half of the sample (n = 11) had a secondary school degree, the majority of the other half (n = 8) a university degree, and most of the participants were retired (n = 15).

**Table 1:** Participants characteristics.

ID	Video	Age range	Gender	Nationality	Education	Occupation
1	Yes	70–74	Male	Swiss	High school	Retired and caregiver
2	Yes	80–84	Male	Swiss	University	Self employed
3	Yes	65–69	Male	Swiss	Apprenticeship	Retired
4A	Yes	75–79	Female	Swiss	High school	Retired
4B	Yes	80–84	Male	Swiss	Apprenticeship	Retired
5	**NO**	60–64	Female	Swiss	Apprenticeship	Self employed
6	Yes	65–69	Female	Swiss	University	Retired and volunteer
7A	Yes	65–69	Male	Italian	High school	Retired
7B	Yes	55–59	Female	Italian	High school	Unemployed
8	Yes	45–49	Female	Swiss	Apprenticeship	Caregiver
9	**NO**	80–84	Female	Swiss	Middle school	Retired
10	Yes	65–69	Male	Swiss	University	Retired
11	Yes	70–74	Male	Swiss	University	Retired
12	Yes	70–74	Female	Swiss	Apprenticeship	Retired and volunteer
13	Yes	75–79	Male	Swiss	Apprenticeship	Retired
14	Yes	85–89	Male	Swiss	University	Retired
15M	Yes	75–79	Female	Swiss	Middle school	Housewife and volunteer
15R	Yes	75–79	Female	Swiss	Middle school	Housewife and volunteer
16	Yes	65–69	Female	Swiss	High school	Retired
17	Yes	60–64	Male	Other	University	Employee
18	Yes	70–74	Male	Swiss	University	Retired
19	Yes	65–69	Female	Swiss	University	Retired

### Emerging themes: Motivations in favour or against participation

The analysis of the interviews elicited themes in relation with two main domains of participation in research: *motivation in favour* and *motivation against* participation. Some participants engaged also in description about their experience of participation.

#### Motivations in favour of participation

Three big themes emerged for the motivations in favour of participation in research: 1. motivations related to the outcomes of research (i.e., scientific benefit); 2. personal motivations (i.e., individual characteristics); and 3. altruistic motivations (i.e., benefit to others). The interviewees had already participated to a previous validation study, therefore the motivations they mentioned in favour retrospectively can be considered as factors related to their participation behaviour. In general, they all showed a positive attitude toward research.

#### Motivations related to the outcomes of research

Participants acknowledged that research is complex, and it needs to follow strict procedures. As some motivations relate to the outcomes of research (Table 2), the premise is that research itself must happen, and this depends on their decision to participate.

**Table 2:** Motivations related to the outcomes of research.

Research requires a lot of data
Advancing knowledge
About the self
About the other
About the illness
Improving the current situation
Improve the life of patients
Improve the life of caregivers
Improve society
Improve in the future
Prevent illness
Slow down the illness/early diagnosis
Sensitize toward the illness

Amongst criteria that determine good research, participants highlighted the need for research to collect large amounts of data in order to gain understanding about the issue at stake. Recognizing that *research requires a lot of data* becomes a driver for participation, as it emerged from the interviewees.

“..on the other hand, I think that.. without these analyses, these procedures that compare thousands of data, it is inconceivable to find solutions that will benefit the single individual.” _(P10)_

The positive attitude towards research was refined towards its outcomes and specifically in terms of its ability of *advancing knowledge*. From the interviews emerged three types of knowledge that are relevant for the expectations of someone who decides to participate: *about the individuals*, *the others*, and *the illness*. Acquiring *knowledge about the self* through research is a strong driver for participation, as one participant puts it:

“In my opinion, it is very informative [to participate] for the ones who join, as we can better figure out what our situation is in comparison to others. We never examine ourselves from the inside: where do I stand?” _(P7B)_

For some participants, it was important that the outcome of research could be to *obtain knowledge about others*, and this was mainly expressed by those who acted as informant during the validation study.

“*Let’s say it was a positive experiment, as my husband was saying*. *Just to test (a bit) his cognitive abilities in this period… Luckily, he is not sick*” *(P7A)*

More generally, an important outcome of research, which drove the behaviour toward participation, was the possibility to *advance knowledge about the illness* which, in the case of dementia, was deemed very serious.

“…if I had Alzheimer, what could I do about that? But if you can, with your study, learn more about Alzheimer’s (disease)..that would be good.. I mean, because this is still an issue nowadays. The study could discover something new” _(P11)_

Another set of motivations in relation to the outcomes of research naturally follows the knowledge acquisition phase. Those are related to the possibility of *improving the current situation*. One important research outcome is the *improvement of the life of patients*, and this expectation moved some of the participants to be involved in the study.

“I really l like studies like this, because Alzheimer’s (disease) is still poorly understood… Alzheimer’s is like a cloud above all of us, it may rain but you do not know if and when you will get wet. If, with your study we could fix a few things, this would be great. Or it could diminish [the disease] even.. I do not know if we will find the magic bullet, but if we could soothe this problem, it would be good. Not dying from Alzheimer would be good. This sentence I have just said is nice! Not dying from Alzheimer would be good.” _(P11)_

In relation to this, some participants underscored the fact that everybody is different, and that research should serve the purpose to improve care by making it possible to respond to patients’ individual needs.

“We all age differently, so I think that we should insist on assisting and caring.” _(P2)_

Some participants extended their concerns to the caregivers of people suffering from dementia; therefore, they saw an opportunity for research to *improve the life of caregivers*. This constituted a motivation for their participation.

“As the disease (i.e. Alzheimer’s) is still incurable, maybe [research] could help with some interventions related to the illness … Help family members, inform family members, shape the environment around these people with dementia and their family.” _(P17)_

Somebody expanded the scope of the improvement to the whole society and underscore the need that research works to *improve society* when it comes to problems such as dementia. Therefore, participants suggested that a strong motivation to take part and to help research is thinking about the broader purpose of contributing to collective improvements at the societal level.

“I could reflect and expand on what I was mentioning… infrastructures, training of people, relatives of people at risk… I don’t have great ideas, I am thinking about it right now… At the political level, we could take some decisions… in order to inform laws, incentives or aids… or in order to save money for it. I don’t know.. we need three billion [euros] for Alzheimer in the next twenty year! Let’s save! But are we sure we will need so much money? _(P17)_

More generally, extensive research on the topic could *improve the future*, and by working on current data could benefit future generations. Someone who participated now can help research to improve the situation in the future.

“I think that the whole society is confronted with problems related to ageing and, among these issues, Alzheimer’s and dementia are among the most important and more recognizable. So, I think that each one of us must contribute at least to a little extent to research, which is fundamental for the future of humanity.” _(P2)_

Medical research was also expected to change something at the level of the illness it focuses on. This was a strong reason for a number of interviewees to participate. *Preventing illness* was one of the expected outcomes.

“Maybe, one day we could prevent Alzheimer’s at a young age, as also people in their sixties suffer from it, maybe after spending all their lives working in front of a computer.. or teachers, people who worked their whole life … Maybe finding a way to postpone or to cure it, maybe not to cure it completely, but to prevent it before knowing that there is the problem.” _(P15)_

Some expressed an expectation towards the outcomes of research in terms of *slowing down the illness/early diagnosis*.

“Research could concentrate on early diagnosis of the illness, and on discovering drugs that are able to slow down this ‘deterioration of the mind’.” _(P5)_

Finally, some participants refer to a side outcome of research as the possibility to *sensitize toward the illness*. This appeared to be critical as it was suggested that dementia awareness is still limited in the public. The decision to participate had to do with improving dementia awareness and reducing stigma.

“I think research is a good thing to raise awareness, to sensitize people, to bring together people with very different situations at home () …Maybe this could encourage people to get involved in volunteering, and help them better understand the problem” (P8)

#### Personal motivations

During the analysis, it emerged that some recurrent personal attitudes and motivations were driving the decision to participate in research (Table 3). One of the most cited was *curiosity*, as many participants expressed in various ways how they decided to participate because of this.

“There is also much curiosity because you take part [in the study] without knowing exactly what to expect, and this makes you curious…” (P7B)

**Table 3:** Personal motivations.

Curiosity
Civic engagement
Interest in the topic
Trust in science
Everyone counts
Openness
Play the game

Another triggering personal motivation was *civic engagement*. Some of our participants underscored the importance to take part in society and the necessity to be available as something that is a civic duty, and some specifically expressed the joy of participating in society.

“I almost always take part in studies because I like to participate in public life, as well as targeted studies….” _(P3)_

For some participants the main personal motivation was their *interest in the topic* of research. The interest may derive for personal experience, but also from direct contacts with people affected by the illness or simply because of personal sensitivity toward such issues.

“I have to say that I have always been interested in problems related to ageing and dementia for various reasons: therefore, it was obvious to me that I had to participate.” _(P2)_

All the participants showed a positive attitude toward research. However, some explicitly expressed a profound *trust in science* and research as their personal reason for choosing to participate in the actual research.

“In any case yes, I trust research a lot. In my opinion, we should invest a lot of money in research because we are moving forwards along this path.” _(P1)_

With respect to research participation some underscored the importance of every single participant, and that research need all of them because *everyone counts*. Some expressed this motivation with quite a pride, as they recognize the value of their own experience into the bigger picture of science.

“Participating in this study means making our library, our life, our reality available, to allow—I may be exaggerating—humanity to grow… But for sure to allow the ones who are conducting studies to collect data that could be used to improve the lives of many people. Maybe even MY life, in a few years…” _(P10)_

In the big theme of personal motivations some specific characteristics of personality were described, and this is the case for *openness*. Some people were happy to recognize themselves open to new experience, and they saw in research participation such an occasion.

“We have always been interested in something new because we do not want to rest on our laurels.” _(P4B)_

This self-perception of participation was also important for others who described their decision in terms of deciding to engage and to *play the game*.

“And we have discussed, we have asked ourselves why not getting tested too? And we were visited by someone here at home and. . . I think it is positive to make yourself available because we often have difficulties such as “no I don’t do that”.. But… Everybody makes himself/herself available based on his/her own capacity…” _(P15M)_

#### Altruistic motivations

The third umbrella theme of motivations to participate in research was altruistic motivations. Among this, three sub themes emerged: the *importance of the contribution*, the *willingness to contribute* and the *willingness to help others* (Table 4).

**Table 4:** Altruistic motivations.

Importance of contribution
Willingness to contribute
Willingness to help others (altruism)

The first theme, the *importance of contribution*, it is per se the premise of the whole altruistic attitude. Recognizing the importance of taking part means taking a first step towards the decision to activate some other altruistic thoughts.

“I find it is important, because the more people join, the more… as you say, you want to reach 100% [of participation]. . . so I would like to contribute, and also to involve other people” _(P3)_

Many participants clearly expressed their main motivations in their *willingness to contribute* to something: research in the specific, an achievement derived from this very research, the work of researchers, a whole societal effort etc.

“It is also a pleasure to contribute to these… With our life experience now, we could contribute to research and give a hand to a work that you have to do” _(P7A)_

Some participants expressed the root of their decision to participate with their *willingness to help others*. Many described themselves as having an altruistic approach in general, in some cases by being involved regularly in voluntary work.

“[I joined] At the beginning to deepen and help this research and to… How do you say that? To help the others, right?” _(P15R)_

#### Motivations against participation

Participants also made an effort in recalling the reasons of their hesitancy, as well as the reasons of somebody who tried to persuade them not to participate, and they even speculated on why people their age may not be willing to participate. These motivations are therefore not directly related to the behaviour of non-participation, but they can be related to the intention of non-participating. The motivation against participation were somehow symmetrical with the motivations in favour and were divided into two main themes: 1. motivations related to the outcomes of research; 2. personal motivations.

#### Motivations related to the outcomes of research

Motivations related to the outcomes of research are listed in Table 5. Some participants reported that if people do not want to take part in research it may be because they have *no interest in knowing*, or because they may be afraid of knowing something about themselves and their health. Knowledge as the outcome of research is not bad *per se*, but it could be for the single person or, even if this is not the case, the person is not interested.

**Table 5:** Motivations related to the outcomes of research (against).

No interest in knowing
Fear
Avoidance

“Here in Switzerland people are closed minded. No-one wants to know, no-one wants to let the others know about themselves, but this is something that should be eradicated, for the sake of science” _(P7B)_

When reflecting about barriers to participation interviewees stated that *fear*, as opposed to curiosity, is a feeling that retain people from participating. And this is also the reason behind not being interested in knowing.

“On the one hand there was curiosity, on the other fear of knowing what they will find out about me…” _(P4A)_

More explicitly, in some cases people are afraid and do not want to know about themselves. This is because they may be familiar with the problem and they fear it, therefore their attitude specifically becomes *avoidance*.

“They [others] do not participate because they have difficulties to be confronted with the topic of memory impairment, because it could already be a problem for them, maybe they have a problem with some family members, and they find it difficult to face this.” _(P12)_

#### Personal motivations

We found seven personal motivations negatively influencing the intention to participate in research are seven (Table 6). The first four are closely related with personality or habits. Some participants clearly stated clearly that it could be because of *closeness* that people do not want to take part.

“Maybe it’s just a personality trait, it’s just that somebody does not want to expose himself or herself.” _(P8)_

**Table 6:** Personal motivations (against).

Closeness
Shyness
Laziness
Being busy
No personal interest
Avoid social commitment
No trust in research

*Shyness* may also be behind non-participation, as people find it difficult to share something about themselves. Some could think that maybe taking part in a research on such topic may make some personal issues emerge, of which they could be ashamed.

*“Maybe they are shy*, *they do not want to express their discomfort”*_(P13)_

It could even be less deep and simply instinctive, as many people may not want to contribute, on the contrary, because they are lazy. *Laziness* is another topic that emerged against participation.

“Other reasons…a bit of laziness…that’s what it is! Not to commit or take responsibilities, as we already have many.” _(P14)_

A practical motivation against participation would be the lack of time. We live in busy times, and even though some participants highlighted that people over 65 should in principle have more time, *being busy* could be a reason for deciding not to participate.

“Lack of time as I said…When did we first plan to meet for this interview? Two months ago?” _(P17)_

Also, interest, be it in research or in the topic, could be reversed as a motivation against participation. Somebody who has *no interest* may simply decide not to participate in a research.

“Maybe indifference… lack of interest, since [some may say] “I am not touched by this problem”, “I am out of this…” _(P6)_

A motivation for taking part in research is civic engagement, and participants reported is a symmetrical motivation against participation, which is *avoid social commitment*.

“In general, it follows the same principle for which half of the citizens do not vote [in general elections]. Even if it is not a big effort, it shows that people are reluctant to participate in public life, to be committed to do something.” _(P3)_

We reported above that those who participated actually had a positive attitude toward research, and some were even motivated by their profound trust in research. However, this could also be the other way around, as *no trust in research* could motivate a decision of non-participation.

“It can be distrust toward the institution or the researchers in general, maybe they believe that scientists want to know too much, or maybe people do not see any point in making themselves available for such a thing.” _(P18)_

## Discussion

We conducted this study to uncover motivations in favour and against participation to an epidemiological study on ageing and dementia impact in older adults. We found a general positive attitude toward epidemiological research in older adults. Our thematic analysis revealed reasonable individual and collective expectations in research across three main motivations (i.e. personal, related to the outcomes of research, and altruistic), in the short-, mid- and long-term. Studying jointly motivations in favour and against provides information for recruitment strategies and to overcome barriers to participation, respectively.

Qualitative inquiry is commonly used to explore the topic of motivation to participate in research [10, 16, 18]. Our findings on the altruistic motive to take part into research are consistent with and extend those of previous studies, which were conducted in younger [19], and in older adults [13, 15–17]. Moreover, our participants’ motivations related to the outcomes of research are consistent with motivations related to the benefits of research for the self and for others, which were previously reported in the field of dementia research [16, 17]. Other studies found that barriers to research participation in dementia research may pertain to practical and logistic reasons also due to reduced mobility and autonomy in old age [20]. Low awareness of research subjects under study, and misperception of the true risks associated to participation have also been reported in older adults [16]. The barriers that emerged in our study pertained to personality traits and fear. Personal factors and characteristics may sketch the “willing participant”. This is novel and potentially relevant to inform recruitment strategies.

Theoretical models posit that intrinsic and extrinsic motivations modulate intentions and behaviours (e.g. Theory of Planned Behaviour; Theory of Reasoned Action) [25, 26]. Our findings are consistent with this dualistic model. Our thematic analysis clearly revealed that motivations in favour and against participation were quasi-symmetrical reflecting the intrinsic (personal) and extrinsic (related to outcomes of research) factors in both directions. However, the altruistic theme did not seem to have a clear counterpart in the motivations against participation in our study. Altruism is one of the facets of the agreeableness personality trait of the five-factor model [27]. Altruism is an intrinsic motive unlikely related to distrust in science or research institutions. Interest in dementia research, and in research as a means to benefit society have been reported in previous studies as key reasons to participate among older adults [9], and can contribute to positively modify distrust. The positive attitude toward participation in epidemiological research that emerged in our study may reflect the trustworthiness of the relationship between participants and scientists. Epidemiological research could build on this. Participatory action research is rooted on mutual trust and early involvement of participants, from conceiving research questions and co-designing studies [10, 28–30], to crafting information materials, including informed consent, so that legitimate and explicit expectations to contribute to research and advancing knowledge are met [13].

The present study has some limitations. Our sample included individuals who already took part in a prior related study. This may have affected their predisposition towards research, and may explain, at least in part, positive attitudes towards research. However, this limitation is shared with other studies [16, 20], because elicitation of information and data from non-participants may be unethical, unpractical, or simply impossible. We cannot exclude that other barriers and other themes related to motivations against participation may exist which did not emerge in our analysis. However, the interviewer built on the retrospective thinking of the interviewees by asking about their reasoning, and about other people’s advice/objection in relation to research participation. Further, the interviewees had participated in a validation not an epidemiological study. The two study designs imply and entail distinct research purposes, and the motivations to participate might not be the same. Nonetheless, we piloted and tested all relevant procedures of the future epidemiological study during the validation study. The participation experience of the two studies is alike because the informed consent, the measures, assessments and procedures are identical. Next, that in half of the interviews a professional video-maker was present in the room, and not only the interviewer, is another potential limitation of our study. We cannot exclude that the presence of a third person may have influenced the participants and the interaction with the interviewer. However, all interviews were semi-structured (i.e. the same questions were asked, and same topics discussed), and conducted by the same, experienced researcher. Moreover, we made several technical adjustments to reduce the intrusiveness of the video-maker in the room who was purposely trained on the research methods and never interfered with the data collection. Finally, although the size of the sample may limit the generalizability of our findings to similar contexts and populations, internal validity was preserved. In qualitative research, sample sizes are confirmed *a posteriori* and determined by saturation or ‘point of redundancy’ [31]. Data saturation was reached and confirmed.

A major strength of this study is that two independent, experienced researchers conducted the in-depth thematic analyses of the interviews. The fact that the interviewer remained aware of the context, the purpose and the focus of research but mastered critical thinking is a further strength of this study because it favoured reflexivity [24], enriched the interview guide, and the development of a true dialogic process. This could shed light on opportunities as well as on systematic procedural mistakes that may hinder the capability of modern research to talk to an older adult population. Our findings have potentially relevant implications for both the design and conduction of epidemiological studies on ageing.

## Conclusion

This study applied a rigorous qualitative procedure to uncover the motivations in favour and against participation in an epidemiological study on ageing and dementia in Switzerland. The ability to delineate and anticipate both favouring and deterring reasons can significantly contribute to attain high and unbiased participation rates in epidemiological studies. Participatory action research studies can inform the design and conduction of epidemiological studies in older adults. Moreover, information on the motivations to participate in and contribute to research should be carefully accounted for and used to optimize community sensitization activities, and the recruitment strategies of participants.

## Supporting information

S1 Appendix Interview grid. (DOCX)
