# Supplementary material for: Older adults’ motivations to participate or not in epidemiological research. Qualitative inquiry on a study into dementia in Switzerland
Source: PLoS One. 2021 Feb 12;16(2):e0247141. doi: 10.1371/journal.pone.0247141 (PMC7880484; doi:10.1371/journal.pone.0247141)
Supplement: S1 Appendix — (DOCX) [file pone.0247141.s001.docx]

S1 Appendix. Interview grid

| Participation to the validation study | |
| --- | --- |
| Participation experience | How was your experience of participating in the validation study?  What did it mean for you to participate in the validation study?  What do you remember from participating? What was the purpose of the study?  How did you feel during the participation?  After participating in the validation study, what expectations did you have concerning the following phases of the project? |
| Motivation to participate | What motivated you to participate in the validation study?  Why do you think other people participate?  Why do you think other people do not participate?  Would you participate if you received an invitation to participate in an epidemiological study on the prevalence of dementia in Switzerland?  [If participant answers “yes” to the previous question] Let us pretend I am one of your friends. Unlike you, I do not want to participate. What would you tell me to convince me? |
| Barriers to participation | Is there anything or someone who made you hesitate with respect to participation?  Was there anything or anyone who pushed you to participate?  In your opinion, what should we do to ensure a high response rate to the epidemiological study? |
| Return of study results | |
| General opinion on the return of study results | Overall, what do you think about the fact that researchers communicate the study results to participants?  What do you think of the fact that researchers report the results of the tests you have been administered? |
| Understanding of the type(s) of study results | What types of results would you be interested to know? |
| Preferences regarding the communication of study results | How would you like the results to be communicated to you, if you agreed to receive them (in writing, verbally)? |
| Preferences regarding who to involve in the communication of the study results | Whom would you want to be informed of your results, if you agreed on the return? |
| Preferences regarding when to communicate the study results | When would you like to be informed of the results, if you agreed on the return? |
| Feelings associated with the return of study results | What feelings come to your mind if you think about having the results communicated to you? |
| Informed consent | |
| General opinion on informed consent | When you participated in the validation study, do you remember if you signed a document?  If so, what do you remember regarding the document you signed?  In general, what do you think about the fact that the participants of a study give their consent to the return of study results? |
| Preferences regarding informed consent procedures | How would you like to provide your consent regarding the return of study results? |
| Preferences regarding who to involve in the informed consent form | Think about the individuals you want to be informed of your study results. Would you include these individuals in the consent? |
| Preferences regarding when to provide informed consent on the return of study results | When would you like to give your consent regarding the communication of the results? |
| Other questions |  |
| Dementia-related challenges | In your opinion, what are the biggest challenges in relation to dementia and Alzheimer's disease?  What and whom should researchers invest on?  What do you expect from research, in general? |
